# Evaluating productive performance, meat quality and oxidation products of Italian White breed rabbits under free-range and cage rearing system

**DOI:** 10.5713/ab.21.0327

**Published:** 2022-01-03

**Authors:** Vincenzo Tufarelli, Alessandra Tateo, Michele Schiavitto, Domenico Mazzei, Giovanna Calzaretti, Vito Laudadio

**Affiliations:** 1 Department of DETO, University of Study of Bari “Aldo Moro”, 70010 Valenzano, Bari, Italy; 2 Department of Veterinary Medicine, University of Study of Bari “Aldo Moro”, 70010 Valenzano, Bari, Italy; 3 Italian Rabbit Breeders Association (ANCI-AIA), 71030 Volturara Appula, Foggia, Italy

**Keywords:** Carcass Traits, Meat Quality, Productive Performance, Rabbit, Rearing System

## Abstract

**Objective:**

Free-range systems have been increasingly available to the consumer due to increased demand for more sustainable meat-products. In the current study, the effect of free-range (FR) and cage system (CS) was explored on growth performance, meat quality and oxidation products in Italian White breed rabbits during the growing-fattening phase (5 to 13 weeks of age).

**Methods:**

Forty rabbits were randomly allotted to two treatment groups according to the rearing system, and each treatment group was replicated five times with four subjects in each replicate (20 rabbits per treatment-group). All rabbits fed the same diet as pelleted, and under FR system, no additional feeds were available to animals.

**Results:**

Rearing system had significant effect on rabbit growth performance, where CS group resulted in higher final body weight (p<0.045) and gain (p<0.029) and better feed efficiency (p<0.025) compared to FR rabbits. Most carcass traits were not affected by rearing system; however, a reduction of abdominal fat content (p<0.015) and meat lipids (p<0.034) was observed in FR rabbits. Rearing system had no effects on meat fatty acid profile, whereas meat from FR rabbits resulted less susceptible to lipid and protein oxidation compared to caged animals.

**Conclusion:**

In overall, FR system could be suggested as a substitute for conventional caged system because of FR system preserved rabbit meat from oxidation.

## INTRODUCTION

Rabbit meat consumption and production in the Mediterranean countries is important, particularly in Italy, France, and Spain; and in the last decades Italy was one of the main producers of rabbit carcasses [1–4]. Moreover, improving rabbit meat quality may encourage people to consume this alternative meat [5–7].

Raising systems have remarkable effects on health [8] and productive traits of rabbits [9]. Alternative systems, such as free-range (FR), coupled with high standards of animal health can improve animal products [10] and meat safety, which is the increasing preference of consumers in many countries [11,12]. Therefore, the interest in obtaining rabbit meat from less intensive rearing systems has increased in the last decade [13], because of the conventional cage production systems have the potential to influence negatively animal production.

Some recently published studies investigated the effects of different rearing systems (as FR, open-air cages, organic farming) on rabbit meat quality and its shelf life [10,14,15] highlighting that the effect of the rearing system has been frequently summed or confused with other factors, such as the presence of a different stocking density and group size [15].

Based on this, the available amount of information about productive performance effects in rabbits raised in alternative systems must be further improved; thus, the aim of this study was to investigate this effect in Italian White breed rabbits.

## MATERIALS AND METHODS

All the experimental procedures were carried out according to the approved protocols by the Institutional Animal Care and Use Committee (IACUC), Department of DETO, University of Bari “Aldo Moro”, Italy (Approval No. 07/2020).

### Animals, diets, and management

The trial was conducted in an experimental rabbit farm located in the province of Bari, Apulia region (Italy), according to the guidelines for applied nutrition experiments in rabbits [16]. A total of 40 male Italian White (Bianca Italiana) breed rabbits, obtained from the Central Breeding Farm of the Italian Rabbit Breeders Association (ANCI-AIA, Volturara Appula, Foggia, Italy) and aged 35 days (body weight 1,045±10.1 g, mean±standard error of the means), were randomly assigned to two groups of 20 animals according to the rearing system: FR and cage system (CS). Within each group, rabbits were divided into five replicates having four rabbits/replicate, for a total of 20 rabbits per group). The rabbits in CS were housed individually under standard conditions between 15°C to 23°C, controlled by heating and forced ventilation systems, in wire cages measuring 360×450×310 mm and at a height of 90 cm from the concrete floor. A cycle of 12-h of light and 12-h of dark was used throughout the experiment. The light was switched on at 08.00 am. The rabbits reared in the FR system had a whole-day access to the range (0800 to 1600 h) and were shepherded to the same house. In the FR system, the available space was 0.25 m^2^/head, so that each area available for replicate having four rabbits was 1 m^2^; also, the FR area was composed by a 3 m high metal fence protected by a shaded net to deny access to predators. Four points of feeding (troughs and nipples water) were supplied in each area under a plastic cover. In the FR area there was no grass but only shelters and trees, so that no supplemental feed was available to rabbits under FR system. Rabbits of both groups were fed *ad libitum* and water was freely available from nipple drinkers. The diet fed to rabbits contained forage as dehydrated alfalfa (*Medicago sativa*) meal previously sieved at 8 mm and formulated to meet or exceed the nutrient requirements of rabbits [2]. Diet was prepared as pelleted form, and pellet quality was determined using a standardized method for durability (method S269.4) [17]. No medication was included in the feed or in the drinking water and rabbits’ health status was checked through individual observations. The ingredients composition and chemical analysis of diet is shown in Table 1.

### Growth performance, carcass traits and meat measurements

From 35 to 91 days of age rabbits were weighed individually at weekly intervals, while the feed intake was daily recorded and the feed to gain ratio was calculated. Mortality of rabbits was equal to zero during the trial. At the end of the fattening period at 91 days of age, 10 rabbits per group were randomly selected in the afternoon for slaughter. On the next morning, the selected rabbits were transferred in small groups to the slaughter facility near the experimental building in the slaughter to determine carcass traits. The rabbits were then weighed (SW), electrically stunned, and slaughtered within 2 h. The slaughtering and carcass dissection procedures followed the World Rabbit Science Association (WRSA) recommendations described by Blasco and Ouhayoun [18]. The stunned rabbits were bled, and then the skin, gastrointestinal tract and the distal part of legs were removed. Carcasses (with head, thoracic cage organs, liver, kidneys, perirenal and scapular fat) were weighed (hot carcass), then chilled at 4°C for 24 h in a ventilated room. After chilling, the chilled carcasses (CC) were weighed. The slaughter yield (CC weight as % of SW) and the ratio of the organs and carcass parts to the CC weight were calculated as required. Immediately after weighing, the intermediate (loin joint) and hind parts were individually packed and cooled at 4°C and transported to the laboratory to determine drip loss (calculated as the 48/24 h post-mortem weight ratio). Meat pH was measured 1 and 24 h *post-mortem* on *Longissimus lumborum* (LL) muscle, using a combined glass penetrating electrode (Ingold, Mettler Toledo, Greifensee, Switzerland). Colour measurements were assessed on the carcass surface over the LL. A chromameter (Minolta CR-300; Minolta, Osaka, Japan) was set to the L* (lightness), a* (redness), and b* (yellowness) scale, as described by Combes et al [19].

### Feeds and meat proximate composition and fatty acid analysis

Feeds and meat samples were analysed in duplicate for dry matter by oven drying method (934.01), total ash by muffle furnace (942.05), crude protein by Kjeldahl method (954.01) and crude fiber (method 973.18) as described by AOAC [20]. Total lipids were extracted according to the method of Folch et al [21]. Neutral detergent fiber, acid detergent fibre, and acid detergent lignin contents were determined according to the sequential procedure of Van Soest et al [22].

In preparation to FA composition analysis, samples of meat (5 g each) were freeze-dried and then ground. Briefly, methyl heptadecanoate (no. 51633; Fluka, St. Louis, MO, USA) was dissolved into n-hexane (1 mg/mL) as an internal standard. Methyl esters of the FA were prepared [23]; samples (300 mg each) and 5 mL of internal standard were incubated (2 h at 80°C) with methanolic acetyl chloride in a total volume of 9 mL. After cooling to room temperature, 7 mL of 7% (wt/vol) K2 CO3 was added with mixing, and then the organic phase was collected after centrifuging at 1,500×*g* for 2 min at 4°C. The FA methyl esters were fractionated over a CP-SIL883 column (100 m×0.25 mm i.d., film thickness 0.20-μm fused silica: Varian, Palo Alto, CA, USA) in a Shimadzu (model 2GC17A; Shimadzu, Kyoto, Japan) gas chromatograph with a Hewlett-Packard HP 6890 gas system (Palo Alto, CA, USA) and using flame ionization detection. Helium was used as the carrier gas at a constant flow rate of 1.7 mL/min. The oven temperature was programmed as follows: 175°C, held for 4 min; 175°C to 250°C at 3°C/min; and then maintained for 20 min. The injector port and detector temperature was 250°C. Samples (1 μL) were injected by an auto-sampler. Output signals were identified and quantified from the retention times and peak areas of known calibration standards. Composition was expressed as percentages of the total fatty acids.

### Meat thiobarbituric acid-reactive substances, protein carbonyls and hydroperoxides analyses

Thiobarbituric acid-reactive substances (TBARS) were determined in meat samples after 7 days of storage at 4°C as described by McDonald and Hultin [24]. Tissue samples (2 g) were weighed into test tubes each with 18 mL of 3.86% perchloric acid; samples were homogenized with a Polytron (IKA Labortechnik T25-B, Selangor, Malaysia) at high speed. Fifty microliters of butylated hydroxyl anisole (BHA) (4.5% BHA in ethanol) were added to the sample prior to homogenization. The homogenate was filtered through a filter paper. The filtrate (2 mL) was mixed with 2 mL of 20 mM TBA in distilled water and incubated in a boiling water bath for 30 min. After cooling, the absorbance of filtrate was determined at 531 nm against a blank containing 2 mL of 3.86% perchloric acid and 2 mL of 20 mM thiobarbituric acid-reactive solution. The TBARS values were expressed as milligrams of malonaldehyde (MDA) per kg of meat.

Meat protein oxidation was evaluated by derivatization with dinitrophenylhydrazine as described by Oliver et al [25] with slight modifications. Briefly, burger patties (1 g) were minced and then homogenized 1:10 (w/v) in 20 mM sodium phosphate buffer containing 6 M NaCl (pH 6.5) using an Ultraturrax homogenizer (IKA-Werke, Staufen, Germany) 2 9 30 s. Two equal aliquots of 0.2 mL were taken from the homogenates and dispensed in 2 mL Eppendorf tubes. Proteins were precipitated by cold 10% trichloroacetic acid (TCA) (1 mL) and subsequent centrifugation for 5 min at 4,200 g. One pellet was treated with 1 mL 2 M HCl (protein concentration measurement) and the other with an equal volume of 0.2% (w/v) dinitrophenylhydrazine in 2 M HCl (carbonyl concentration measurement). Both samples were incubated for 1 h at room temperature. Afterwards, samples were precipitated by 10% TCA (1 mL) and washed three times with 1 mL ethanol:ethyl acetate (1:1, v/v) to remove excess dinitrophenylhydrazine. The pellets were then dissolved in 1.5 mL of 20 mM sodium phosphate buffer containing 6 M guanidine HCl (pH 6.5), stirred and centrifuged for 2 min at 4,200 *g* to remove insoluble fragments. Protein concentration was calculated from the absorption at 280 nm using bovine serum albumin as the standard. The amount of carbonyls was expressed as nmol of carbonyl per mg of protein using an absorption coefficient of 21.0 nM-1 cm-1 at 370 nm for protein hydrazones.

For hydroperoxides analysis, meat samples (2 g) were ho mogenized in 20 mL of 0.15 M KCl for 2 min. Two aliquots of homogenate (50 μL each) were added with 1 mL 100 μL/mL TCA and then centrifuged at 1,200×g for 3 min at 4°C to measure protein oxidation. The first aliquot was used as a standard and added with 1 mL of 2 M HCl solution. The second aliquot was added with 1 mL of 2 M HCl containing 10 mM 2,4-dinitrophenyl hydrazine (DNPH). Samples were incubated for 1 h at room temperature (15°C to 30°C) and shaken every 20 min, and then 1 mL of 100 μL/mL TCA was added. The samples were vortexed for 30 s and centrifuged 3 times at 1,200×*g* for 3 min at 4°C and the supernatant removed. Care was taken not to disrupt the pellet. The pellet was washed with 1 mL of ethanol:ethyl acetate (1:1), shaken, and centrifuged 3 times at 1,200×*g* for 3 min at 4°C and the supernatant removed. The pellet was then dissolved in 1 mL 20 mM sodium phosphate 6 M guanidine hydrochloride buffer. Samples were then shaken and centrifuged at 1,200×g for 3 min at 4°C. Carbonyl concentration was calculated on the DNPH treated sample at 360 nm with a Beckman Coulter DU800 (Beckman Instruments Inc., Brea, CA, USA) and expressed as nanomoles carbonyl per milligram protein. Protein concentration was calculated according to the Biuret assay [26,27].

### Statistical analysis

Data from growth and slaughter trial were analysed by one-way analysis of variance using the general linear model procedure of SAS Institute Inc. [28]. Each replicate within treatment was considered as experimental unit. Data are presented as least-squares means and the difference among means was tested by Tukey’s test. A level of p<0.05 was used as the criterion for statistical significance.

## RESULTS AND DISCUSSION

Current trends show that the demand for rabbit meat will increase in the near future [6,7]. With consumers’ interest in healthy products and animal-friendly production systems, free range production system could play a key-role on meat marketability, including rabbit meat.

The effect of dietary treatments on growth performance is shown in Table 2. No rabbits died during the trial and there was an optimal consumption of the diets in both groups. The final live weight, daily weight gain of caged rabbits were slightly heavier than FR (p = 0.045) and had higher average daily gains (p = 0.029). Rabbits under FR system consumed more feed compared to caged animals (129 vs 123 g/d; p = 0.041). Feed to gain ratio differed significantly (p = 0.025) according to the rearing system, where caged rabbits resulted in lower ratio. Similar growth performance including feed to gain ratio were also reported in earlier studies with rabbits under different rearing systems [1,10,12,15]; however, the obtained findings were satisfactory and in line with the requested fattening standards of rabbits. For example, Castellini et al [29] showed that conventionally raised broilers to have higher growth rates than those raised in outdoor systems. Moreover, according to Sarica et al [30], weight gain of outdoor birds is lower than that of those in a controlled environment because of fluctuating weather conditions and increased opportunities for exercise. This trend may reflect the results obtained on rabbits reared under caged or FR systems. However, both free-range and caged rabbits in this study appear to have completed the fattening period within 15 weeks of age, given the similarities of weights and the relatively increases in body weight observed during this time period.

Furthermore, variations in the feed formulation and in gredients inclusion level in rabbit diet appeared to be the main cause of inconsistencies in animals’ performance as found in the available literature [6,31]. The changing of rearing system from cage to FR system is a tendency aiming to attend the best conditions of animal welfare. In the present study, it was verified that rabbits reared in FR system did not show any difference in most of carcass traits and meat parameters compared to those reared in CS (Table 3). The rearing system has significantly influenced some of these parameters. Slaughter carcass weight was slightly different (p = 0.044) in the two systems (2.5 vs 2.4 kg, caged vs free-range, respectively). This finding agrees with other recently published studies [10,12,15] examining the effect of space allowance and rearing system on rabbit carcass characteristics. In our study, slaughter yield did not vary among groups (p>0.05), indicating that raising rabbits under FR system did not depress carcass yield. This difference may be due to the breed, age, amount of fat in diet and the number of internal organs left with the rabbit carcass after slaughter. It was interesting to note that rabbits under FR system presented a significantly lower (p = 0.015) proportion of abdominal fat (on % of CC) compared to caged animals. Similarly, non-caged system had a positive effect (p = 0.027) on meat water-holding capacity resulting in lower retention than carcasses of caged rabbits. Differences in abdominal fat ratios by production system could be explained by the higher body weights of the caged rabbits and the greater physical activity of the FR animals.

The values of pH on LL muscle determined 1 h and 24 h after slaughtering were not significantly influenced by treatments. Conversely, Dalle Zotte et al [32] found significantly lower pH in rabbit meat, maybe due to a feed rationing during the post-weaning phase. The same authors found that, in some cases, it was observed that as age, as well as weight, increased, the glycolytic energy metabolism also increased and correlatively, the oxidative metabolism and pH decreased.

Meat colour parameters showed significant differences due to the rearing system in both considered muscles. The LL muscle showed a significant and higher redness (a*) (p = 0.014) and yellowness (b*) (p = 0.009) values in rabbits under FR, different from those obtained by Lazzaroni et al [33] who observed the same colour parameter as function of housing systems. On the contrary, the values observed on both muscles in lightness (L*) were not confirmed by significant differences. The latter result was not in agreement with the same authors who reported higher values for male rabbits and therefore a different coloured muscle compared to females. Moreover, the differences in meat colour measurements found in this work compared to the cited authors could be due to different age at slaughter varying from 90 up to 110 days of age. Nevertheless, these values were in the normal range and therefore this muscle would not be considered to be excessively dark for rabbit [3].

Considering the meat proximate composition, differences in mean meat moisture, protein, or ash percentage of rabbits under the two rearing systems were not different (Table 3), but FR rabbits exhibited a reduction in meat fat content compared to caged animals (p = 0.034). Our findings were consistent with previous reports where rabbits [12,15] were reared under different rearing systems (caged or FR).

The effect of rearing system on fatty acid composition of rabbit meat is presented in Table 4. Meat from *Longissimus lumborum* muscle of FR rabbits exhibited a significant lower fat content, confirming the optimal nutritional value of meat produced by animals under alternative systems. The fatty acid content of human foods has become increasingly important, as several fatty acids have been implicated in health issues in humans [34–36]. In this study, we attempted to influence the content of rabbit meat fatty acid known to be of detrimental to human health. Meat from rabbits under both rearing systems had similar amounts of total saturated fatty acids, monounsaturated fatty acids, polyunsaturated fatty acids, as well as the level of n-6 and n-3 polyunsaturated fatty acids. The n-6/n-3 ratio were also similar between the two groups. Therefore, alternative and friendly rearing systems did not negatively affect the meat fatty acids profile of rabbits. It was interesting to note that rearing system had significant effect on meat oxidation products. The meat from FR rabbits had a lower thiobarbituric acid-reactive substances level compared to caged rabbits (p = 0.0027). These results could be partially explained by the low lipid/ and heme iron contents in *Longissimus lumborum* than in other muscles [36]. The heme and non-heme iron present in meat can be considered as prooxidants because of their reaction with hydroperoxide to initiate lipid oxidation in meat and meat products [33,37]. However, the level of thiobarbituric acid-reactive substances considered as the threshold for perception and acceptability of oxidation by consumer depends on the species. The concentrations of lipid hydroperoxides muscle did not differ (p>0.05) among treatments. A significant effect on protein oxidation (p = 0.031) related to the rearing system in meat was observed. The carbonyl levels resulted to be higher in rabbits under conventional CS compared to FR animals (1.92 vs 1.62 nmol of dinitrophenylhydrazine/mg protein, respectively). The protein carbonyl content was used as a measure of the extent of oxidative reactions affecting muscle proteins during storage of meat patties. Carbonyl compounds are formed as a result of the oxidative degradation of side chains of lysine, proline, arginine and histidine residues [38]. The level of protein carbonyls in the present study indicates optimal oxidative reactions. Our finding also confirmed the results of recently published literature investigating the effect of alternative rearing systems on rabbit meat stability [12,15]. Moreover, protein oxidation seems to be influenced by the level of lipid oxidation in meat [37]. The levels of thiobarbituric acid-reactive substances observed in the present study were correlated with carbonyl proteins levels.

## CONCLUSION

The changing of rearing system from cage to FR system is a tendency aiming to attend the best conditions of livestock species, including rabbits. In the present study, it was assessed that rearing rabbits under FR system supported positively the meat quality compared to caged rabbits, despite a slight reduction in growth performance. Moreover, an improvement of meat oxidative stability was verified under animal-friendly production system. Thus, FR system could be suggested in rabbits as a substitute for conventional caged system; however, further studies are needed to evaluate more welfare-related indices in rabbits under alternative rearing systems.

## Figures and Tables

**Table 1:** Ingredients and chemical composition of the diet fed to rabbits

Items	
Ingredients (g/kg diet)	
Dehydrated alfalfa meal	285
Dehydrated beet pulp	285
Corn	200
Soybean meal, 48% crude protein	100
Wheat middlings	84.5
Cane molasses	20
Vitamin-mineral premix^1)^	50
Monocalcium phosphate	50
Sodium chloride	40
Calcium propionate	25
L-lysine	25
DL-methionine	25
Yeast	10
Magnesium oxide	10
Magnesium carbonate	10
Chemical composition (g/kg as-fed)	
Dry matter	891
Crude protein	154
Ether extract	24
Crude fiber	141
NDF	268
ADF	167
ADL	39
Ash	69
Digestible energy (MJ/kg)^2)^	10.61

NDF, neutral detergent fiber; ADF, acid detergent fibre; ADL, acid detergent lignin.

1) Provided per kg of diet: vitamin A 12,500 IU; vitamin D_3_ 1,500 IU; vitamin E 30 mg; vitamin B_1_ 1.5 mg; vitamin B_2_ 5 mg; vitamin B_6_ 2 mg; vitamin B_12_ 0.02 mg; vitamin PP 20 mg; vitamin K_3_ 2.5 mg; folic acid 0.75 mg; pantothenic acid 10 mg; D-biotin 0.1 mg; choline chloride 300 mg; MnSO_4_ 150 mg; FeSO_4_ 5 mg; ZnSO_3_ 75 mg; CuSO_4_ 5 mg; KI 1 mg; CoSO_4_ 0.2 mg; Na_2_SeO_3_ 0.1 mg.

2) Calculated as: 12.912–(0.0236×crude fiber)+(0.010×crude protein)+(0.020 ×ether extract) [10].

**Table 2:** Effect of rearing system on growth performance of rabbits

Item	Conventional cage	Free-range	SEM	p-value
No. of rabbits	20	20		
Initial BW at 35 d (g)	1,050	1,062	21.22	NS
Final BW at 91 d (g)	2,571	2,425	40.15	0.045
BW gain (g/d)	27.1	24.2	2.51	0.029
Feed intake (g/d)	123	129	8.66	0.041
Feed to gain ratio	4.54	5.33	0.07	0.025
Mortality (%)	0	0	-	-

SEM, standard error of the means; BW, body weight; NS, not significant.

**Table 3:** Effect of rearing system on carcass traits, colour and proximate composition of meat muscle (Longissimus lumborum, LL) of rabbits

Item	Conventional cage	Free-range	SEM	p-value
No. of rabbits	10	10		
Traits (g)				
Slaughter weight	2,503	2,410	85.35	0.044
Hot carcass	1,505	1,472	31.67	NS
Chilled carcass	1,433	1,365	27.58	NS
% Slaughter weight				
Slaughter yield	60.1	61.1	0.95	NS
Skin	15.5	15.9	0.10	NS
% Chilled carcass				
Heart+liver+lungs	7.11	7.32	0.15	NS
Kidneys	1.39	1.42	0.07	NS
Digestive tract	14.5	14.9	0.12	NS
Abdominal fat	1.21	1.01	0.08	0.015
WHC (%)	17.46	19.87	1.05	0.027
pH_1_ LL	6.62	6.67	0.04	NS
pH_24_ LL	6.52	6.55	0.03	NS
Colour of LL muscle				
L*	42.88	41.26	0.702	NS
a*	5.37	7.27	0.443	0.014
b*	−3.21	−2.13	0.215	0.009
Proximate composition (%)				
Moisture	72.9	72.7	0.093	NS
Protein	22.4	22.6	0.125	NS
Lipids	1.25	1.10	0.068	0.034
Ash	1.52	1.39	0.087	NS

SEM, standard error of the means; NS, not significant; WHC, water-holding capacity.

**Table 4:** Effect of rearing system on meat fatty acid composition (% FAME) and oxidation products of rabbits

Item	Conventional cage	Free-range	SEM	p-value
No. of rabbits	10	10		
Fatty acids (%)				
∑SFA^1)^	38.32	38.75	0.77	NS
∑MUFA^2)^	35.57	36.01	0.81	NS
∑PUFA^3)^	26.11	25.24	0.69	NS
n-6 PUFA^4)^	25.92	26.13	0.41	NS
n-3 PUFA	6.05	6.28	0.27	NS
n-6/n-3^5)^	4.28	4.16	0.15	NS
Meat oxidation				
TBARS (mg MDA/kg of meat)	0.47	0.34	0.025	0.027
Hydroperoxides (μmol/g of meat)	0.33	0.32	0.031	NS
Protein carbonyls (nmol DNPH/mg protein)	1.92	1.62	0.074	0.031

SEM, standard error of the means; NS: not significant; SFA, saturated fatty acids; MUFA, monounsaturated fatty acid; PUFA, polyunsaturated fatty acid; TBARS, thiobarbituric acid reactive substances; MDA, malonaldehyde; DNPH, 2,4-dinitrophenyl hydrazine.

1) Sum of all even chain fatty acid up to 22:0.

2) Sum of 14:1, 16:1, 18:1, 20:1 and 22:1.

3) Sum of 18:2, 18:3, 20:2, 20:3, 20:4, 20:5, 22:4, 22:5 and 22:6.

4) Sum of 18:2, 18:3 n-6, 20:2, 20:3 n-6, 20:4 and 22:2.

5) Sum of 18:3 n-3, 20:3 n-3, 20:5, 22:5 and 22:6.
